# The prevalence and clinical features of pulmonary embolism in patients with AE-COPD: A meta-analysis and systematic review

**DOI:** 10.1371/journal.pone.0256480

**Published:** 2021-09-02

**Authors:** Xiaofang Fu, Yonghong Zhong, Wucheng Xu, Jiangang Ju, Min Yu, Minjie Ge, Xiaofei Gu, Qingqing Chen, Yibo Sun, Huaqiong Huang, Linfeng Shen

**Affiliations:** 1 Department of Respiratory, First People’s Hospital of Yuhang, Hangzhou, China; 2 Key Laboratory of Respiratory Disease of Zhejiang Province, Department of Respiratory and Critical Care Medicine, Second Affiliated Hospital of Zhejiang University School of Medicine, Hangzhou, Zhejiang, China; Beaumont Hospitals: Beaumont Health, UNITED STATES

## Abstract

**Background:**

The prevalence of pulmonary embolism (PE) in the acute exacerbation of chronic obstructive pulmonary disease (AE-COPD) is highly controversial. We conducted a systematic review and meta-analysis to summarize the epidemiology and characteristics of PE with AE-COPD for current studies.

**Methods:**

We searched the PubMed, EMBASE, Cochrane Library and Web of Science databases for studies published prior to October 21, 2020. Pooled proportions with 95% confidence intervals (95% CIs) were calculated using a random effects model. Odds ratios (ORs) and mean differences (MDs) with 95% confidence intervals were used as effect measures for dichotomous and continuous variables, respectively.

**Results:**

A total of 17 studies involving 3170 patients were included. The prevalence of PE and deep vein thrombosis (DVT) in AE-COPD patients was 17.2% (95% CI: 13.4%-21.3%) and 7.1% (95% CI: 3.7%-11.4%%), respectively. Dyspnea (OR = 6.77, 95% CI: 1.97–23.22), pleuritic chest pain (OR = 3.25, 95% CI: 2.06–5.12), lower limb asymmetry or edema (OR = 2.46, 95% CI:1.51–4.00), higher heart rates (MD = 20.51, 95% CI: 4.95–36.08), longer hospital stays (MD = 3.66, 95% CI: 3.01–4.31) were associated with the PE in the AE-COPD patients. Levels of D-dimer (MD = 1.51, 95% CI: 0.80–2.23), WBC counts (MD = 1.42, 95% CI: 0.14–2.70) were significantly higher and levels of PaO_2_ was lower (MD = -17.20, 95% CI: -33.94- -0.45, *P*<0.05) in the AE-COPD with PE group. The AE-COPD with PE group had increased risk of fatal outcome than the AE-COPD group (OR = 2.23, 95% CI: 1.43–3.50).

**Conclusions:**

The prevalence of PE during AE-COPD varies considerably among the studies. AE-COPD patients with PE experienced an increased risk of death, especially among the ICU patients. Understanding the potential risk factors for PE may help clinicians identify AE-COPD patients at increased risk of PE.

**Prospero registration number:**

CRD42021226568.

## Background

Chronic obstructive pulmonary disease (COPD) is a chronic inflammatory condition of the respiratory tract and the third leading cause of morbidity and mortality in the worldwide [1–4]. The prevalence of spirometry-defined COPD was 8.6% in Chinese adults over 20 years old during our study period, accounting for 99.9 million people [5].

People with COPD sometimes experience a worsening of their symptoms, known as acute exacerbation. Although an exacerbation is not necessarily inevitable for COPD patients, it is common. The consequences of pulmonary embolism in these patients with an acute exacerbation of COPD may be devastating [6]. A recent study identified AE-COPD as an independent risk factor for PE [7, 8] and revealed that the occurrence of PE in patients with COPD was approximately four-fold higher than that in patients without COPD [9]. PE is associated with increased mortality in patients with COPD compared to the general population [10]. According to a recent review, the prevalence of PE was approximately 16.1% (range 3.3%-29.1%) in unexplained AE-COPD patients [6]. Based on the results, the authors concluded that PE should receive increased attention in patients with unexplained AE-COPD. However, because of the limited sample sizes in the studies considered in that review, the exact prevalence of PE in AE-COPD remains unknown.

The clinical manifestations of AE-COPD include a sudden deterioration of respiratory symptoms and respiratory function, which are quite similar to PE [11–13]. Identifying AE-COPD patients combined with PE is a challenge because it is difficult to determine whether signs and symptoms are caused by AE-COPD, PE, or both [14]. There is evidence that PE may be common in acute exacerbations of COPD in previous systematic reviews. Rizkallah *et al*. [15] revealed that one of four AE-COPD patients who require hospitalization may have PE. Aleva *et al*. [6] not only described the high incidence of PE among AE-COPD patients but also summarized the localization of thrombi, possible treatments and the prognostic significance of PE among AE-COPD patients. However, there is some discrepancy among studies in the risk factors for increased PE in patients with AE-COPD [6, 14]. Although the indicators employed in the abovementioned studies are quite comprehensive, clinical studies of AE-COPD combined with PE have been increasing in recent years.

Hence, in this study, we systematically pooled data to update the prevalence of PE among those with AE-COPD patients and describe its epidemiology and characteristics, which may be conducive to rapid diagnosis by clinicians.

## Method

### Search strategy and selection criteria

Our study was conducted in accordance with the Preferred Reporting Items for Systematic Reviews and Meta-Analyses (PRISMA) guidelines [16] and the protocol was registered with PROSPERO (CRD42021226568). For this systematic review and meta-analysis, we searched the PubMed, EMBASE, Cochrane Library, and Web of Science databases for articles published from inception to October 21, 2020, using the following search terms: “chronic obstructive pulmonary disease”, “chronic airflow obstruction”, “chronic obstructive lung disease”, “pulmonary embolism”, “pulmonary thromboembolism” and related terms. The full list of the search strategy is shown in S1 Table. In this systematic review, there was no restriction regarding study designs. Studies with reported PE prevalence in AE-COPD or with sufficient data (e.g., sufficient number of positive PE cases and sample size) to calculate the PE prevalence rate in AE-COPD patients were included. We excluded articles with duplicate publication data, case reports, reviews and studies without original data.

### Screening, data extraction and quality assessment

The references of eligible studies were screened, and two reviewers (XF and YH) independently reviewed all citations that met the inclusion criteria. Study selection was performed in two stages: first, the titles and abstracts were screened; second, the full texts were reviewed. Any disagreements regarding eligibility were resolved by consensus. Data from eligible studies were extracted into a database using Microsoft Excel 2016. We extracted the following data: (a) characteristics of studies (title, first author, journal name, country of study population, year of publication, study design); (b) characteristics of participants (numbers in each group, demographics, comorbid illnesses, laboratory data, clinical features). Data extraction was performed by two independent investigators (XF and MJ), and discrepancies were resolved by consensus.

The quality of all studies was assessed using the Newcastle-Ottawa quality scale (NOS) for nonrandomized trials and observational studies, which is recommended by the Agency for Healthcare Research and Quality [17]. This scale consists of 11 items for evaluating studies in terms of information source, inclusion and exclusion criteria, study period, selection of participants, blinding, quality assurance, possible confounding variables, handling of missing data, participants’ response rates, and completeness of data collection. Each item was assigned 1 point if the condition was fulfilled, and the highest point was 11. All included articles are divided into 3 levels according to the total score, which are 0–3 points, 4–7 points, and 8–11 points, respectively, with low, medium, and high-quality scores. The articles and citations were managed in Endnote (version X7).

### Data analysis

Some characteristics of participants are presented as the median and interquartile range (IQR) or median and range in the original papers; the ranges provided were converted to means and standard deviations (SDs) by using an online tool (http://www.comp.hkbu.edu.hk/~xwan/median2mean.html) developed by Dehui Luo. Then, meta-analysis prevalence estimates were transformed using the Freeman-Tukey double arcsine transformation for stabilizing the variance [18], which is preferred over the logit transformation [19]. We estimated heterogeneity among studies with the I^2^ test, which describes the percentage of variation between studies that is due to heterogeneity rather than chance [20, 21]. If I^2^ > 50%, the heterogeneity across studies was significant, and a random effects model was used in the meta-analysis; otherwise, a fixed effects model was used. After pooling the estimates, the combined point estimates and their 95% confidence intervals (CIs) were then back-transformed to proportions and plotted. Funnel plots and Egger weighted regression were used to assess the publication bias [22]. The analyses were performed using R version 3.2.3 (R Foundation for Statistical Computing) and the Cochrane Collaboration Subscription Software RevMan 5.3.

## Results

### Search results

**Fig 1:**
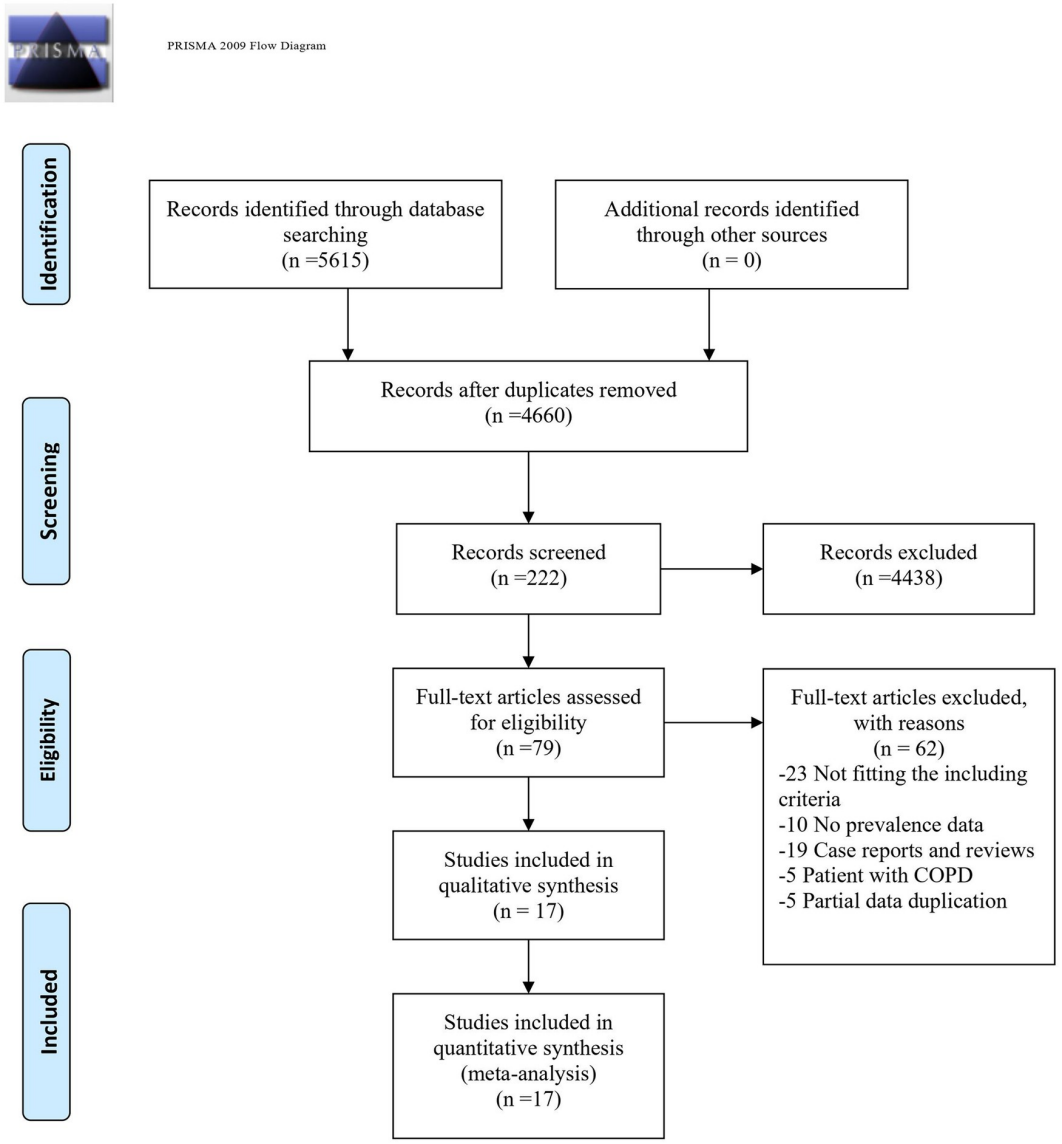
Flow diagram of the study selection process for the meta-analysis. *From*: Moher D, Liberati A, Tetzlaff J, Altman DG, The PRISMA Group (2009). *P*referred *R*eporting *I*tems for *S*ystematic Reviews and *M*eta-*A*nalyses: The PRISMA Statement. PLoS Med 6(7): e1000097. doi:10.1371/journal.pmed.1000097**For more information, visit**www.prisma-statement.org.

Our literature search yielded 5615 records. After the removal of 955 duplicates, we checked the titles and abstracts of 4660 records, and 222 articles remained. After further screening, we removed 143 articles because of irrelevance. The full texts of 79 articles were independently assessed based on the inclusion criteria. Ultimately, the meta-analysis study included 17 studies [6, 10, 23–37] with sufficient data to calculate PE prevalence in AE-COPD patients published between 2010 and 2020. Details regarding the reasons for study exclusion are provided in Fig 1, and the characteristics of the included studies are summarized in S2 Table.

The NOS was used to assess the quality of all 17 studies: 5 were identified as high quality, and 12 were identified as medium quality (S2 and S4 Tables).

### The prevalence of PE and DVT in AE-COPD

**Fig 2:**
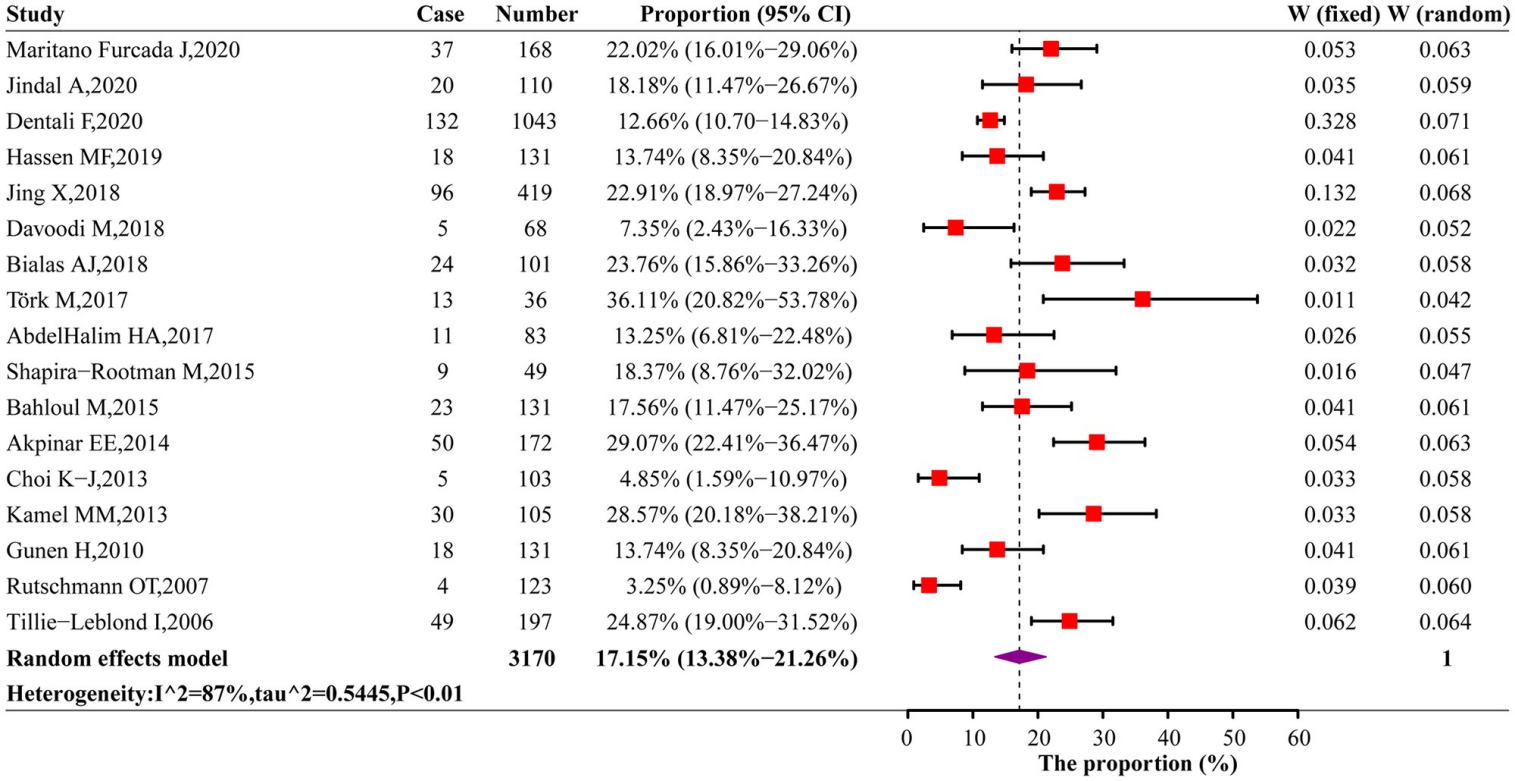
Forest plots of the PE prevalence in AE-COPD patients.

A total of 3170 individuals were identified, 544 of them were positive for PE. Due to the heterogeneity, we used a random effects model to estimate the prevalence of PE and DVT in AE-COPD patients. The pooled PE prevalence in AE-COPD patients was 17.2% (95% CI: 13.4%-21.3%). The highest and lowest prevalence of PE in AE-COPD patients occurred in Turkey (36.1%; 95% CI: 20.8%-53.8%) [29] and Switzerland (3.3%; 95% CI: 0.9%-8.1%) [36], respectively (Fig 2, S2 Table). The pooled DVT prevalence among AE-COPD patients was 7.1% (95% CI: 3.7%-11.4%) (S1 Fig).

### Subgroup analysis of PE prevalence in AE-COPD

**Fig 3:**
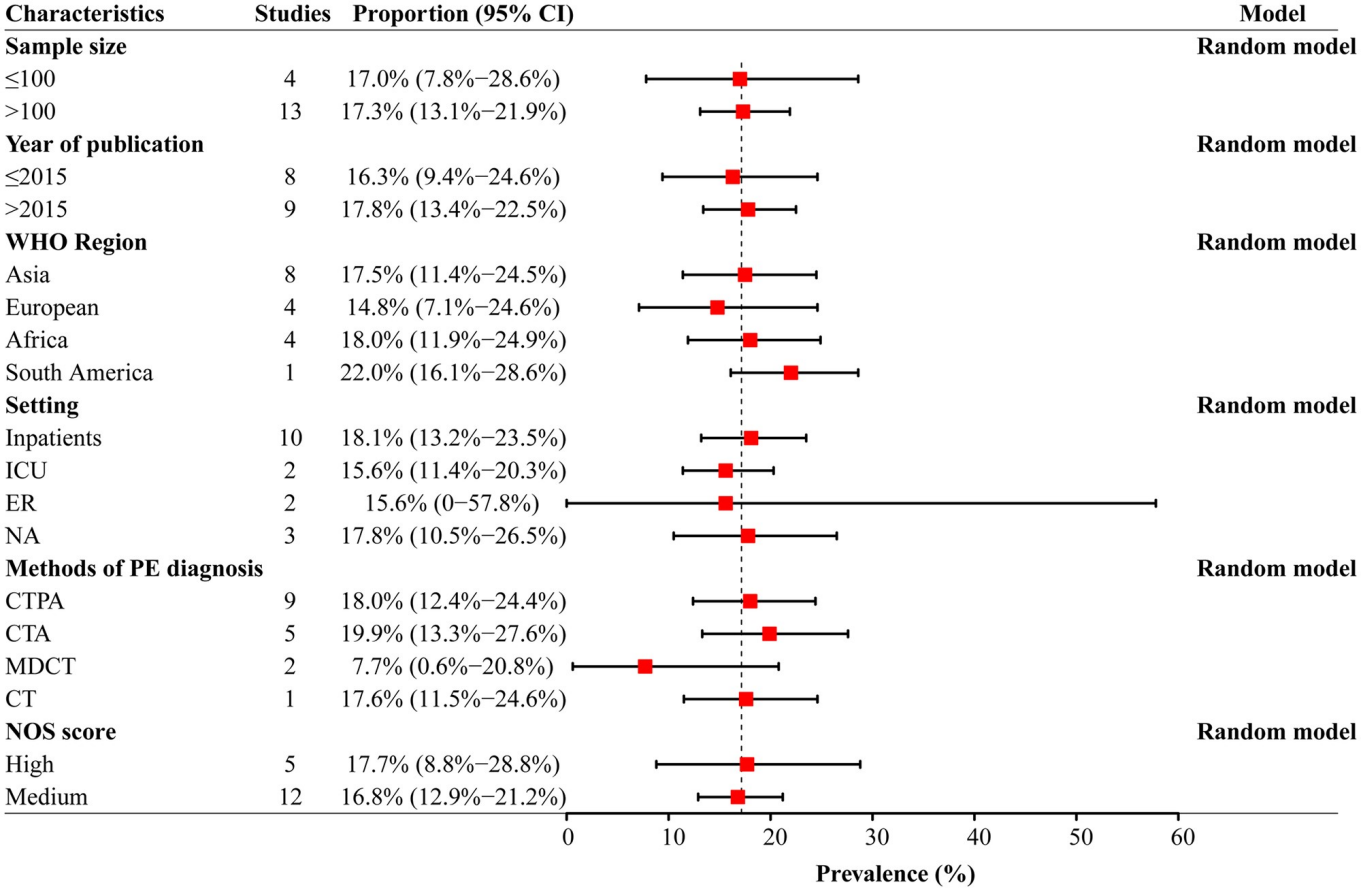
Subgroup analysis of PE prevalence in AE-COPD patients.

The subgroup meta-analysis of PE prevalence in AE-COPD patients is shown in Fig 3. PE prevalence estimates were higher in studies published after 2015 years (17.8%, 95% CI: 13.4–22.5%). The highest PE prevalence rate was in the South American region (22.0%, 95% CI: 16.1%-28.6%); followed by the Africa (18.0%, 95% CI: 11.9%-24.9%) and Asia (17.5%, 95% CI: 11.4%-24.5%). The lowest PE prevalence rate was in the Europe (14.8%, 95% CI: 7.1%-24.6%). The prevalence of PE in hospitalized patients with AE-COPD (18.1%, 95% CI: 13.2%-23.5%) was highest than among patients in the emergency room (15.6%, 95% CI: 0–57.8%) and ICU (15.6%, 95% CI: 11.4%-20.3%).

### Subgroup analysis of the clinical characteristics

As shown in S3 Table, the female gender was a risk factor for the AE-COPD with PE group (OR = 1.33, 95% CI: 1.06–1.67, *P*<0.05). PE should be taken into account when AE-COPD patients had dyspnea (OR = 6.77, 95% CI: 1.97–23.22, *P*<0.01), pleuritic chest pain (OR = 3.25, 95% CI: 2.06–5.12, *P*<0.01), lower limb asymmetry or edema (OR = 2.46, 95% CI: 1.51–4.00, *P*<0.01), higher heart rate (MD = 20.51, 95% CI: 4.95–36.08, *P*<0.01), and longer hospital stays (MD = 3.66, 95% CI: 3.01–4.31, *P*<0.01). Age, smoking, BMI or comorbidities (hypertension, heart failure (HF), atrial fibrillation (AF), ischemic heart disease (IHD), diabetes, cancer, and immobilization) did not increase the risk of PE in patients with AE-COPD.

**Fig 4:**
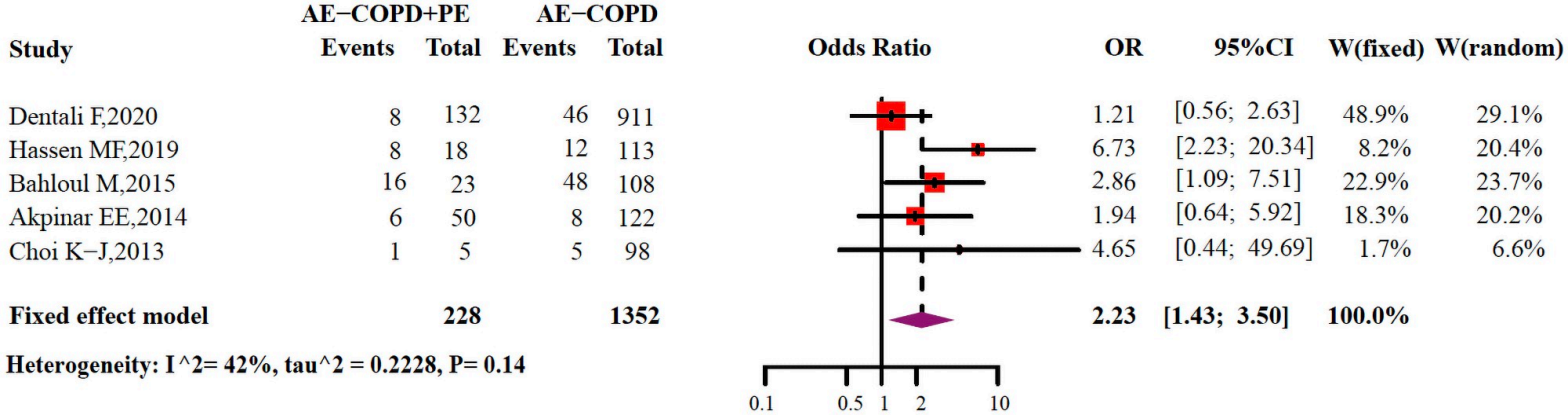
Meta-analysis of studies reporting mortality data.

Five of the included studies reported the mortality rate of PE with AE-COPD [24, 25, 32, 33, 35]. Fatality in the AE-COPD with PE group was significantly higher than the AE-COPD groups (17.1% vs. 8.8%, *P*<0.01). The AE-COPD with PE group had increased the risk of death than the AE-COPD group (OR = 2.23, 95% CI: 1.43–3.50), and a low level of heterogeneity was observed (*I*^2^ = 42%; Fig 4). The mortality rates of AE-COPD patients with PE in the ICU setting were 69.6% [25] and 44.4% [32], respectively, which were significantly higher than those in the inpatient setting (Fig 4).

### Subgroup analysis of the laboratory testing

As shown in S2 Fig, a significant trend towards higher D-dimer (MD = 1.51, 95% CI: 0.80–2.23) and WBC counts (MD = 1.42, 95% CI: 0.14–2.70) in the AE-COPD with PE group. However, AE-COPD patients with PE had lower levels of PaO_2_ (MD = -17.20, 95% CI: -33.94- -0.45, *P*<0.05). In addition, the laboratory tests of PH, PaCO_2_, FEV1 and CRP revealed no significant difference between the AE-COPD patients with PE and the AE-COPD patients. Furthermore, a random-effects model was utilized to analyses laboratory testing because there was obvious sample heterogeneity among the studies except for WBC counts level.

### Publication bias

**Fig 5:**
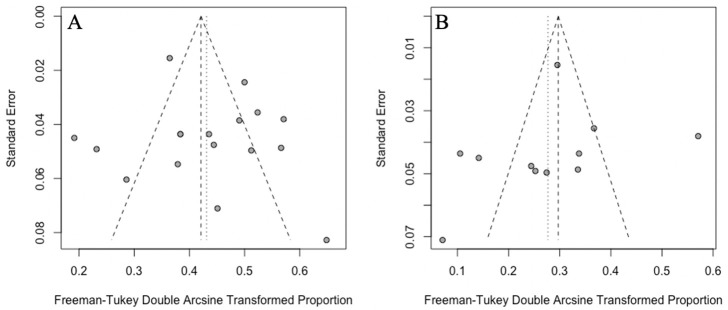
Funnel plots assessing publication bias. A: Funnel plot assessing publication bias in studies reporting the prevalence of PE in AE-COPD patients; B: Funnel plot assessing publication bias in studies reporting the prevalence of DVT in AE-COPD patients.

The shapes of the funnel plots were relatively symmetric (Fig 5). Egger’s test was also conducted to assess publication bias in studies reporting the prevalence of PE and DVT prevalence in AE-COPD patients respectively, and the p-values were 0.5081 and 0.5796 (*P*> 0.05), respectively, which indicated no obvious publication bias.

## Discussion

Our study revealed that the pooled PE prevalence among 3170 AE-COPD patients was 17.2%, which is slightly higher than estimated prevalence of 16.1% found in systematic review from 2017 that included 880 patients. [6]. The prevalence of PE among AE-COPD patients varied widely among our included studies, from 3.3%~36.1%. Consistent with previous systematic reviews [6, 15], our study revealed that the incidence of DVT among AE-COPD was lower than that of PE. Five studies were excluded from our original analyses because they enrolled patients with COPD and not those with AE-COPD [38–42]. Including these five studies in the analysis yields a slight increase in PE prevalence in COPD patients (19.9%; 95% CI, 15.2%-25.7%). Previous studies have shown that PE prevalence is much higher in patients with COPD than in other medical inpatients [43]. Inpatients with COPD are at increased risk of developing venous thromboembolism (VTE) due to acute exacerbations [14, 44–46]. 50%-75% of VTE events, including fatal PE, occur during medical service hospitalization, and it is important for medical staff to prevent and treat VTE in the early stage [47]. However, the reported prevention rate of VTE in patients with AE-COPD is 26.6% [48, 49].

A systematic review from 2017 of PE in patients with AE-COPD reported a prevalence of 16.1% and included studies published before 2015 [6]. However, the PE prevalence estimated based on studies after 2015 was higher than that from studies published before 2015. Owing to the recent advancements in CT imaging, the accuracy and sensitivity of PE diagnosis have greatly improved. In addition, clinicians are more alert than they were in previous years to patients with clinical symptoms such as dyspnea. Our study suggested that the pooled prevalence of PE among AE-COPD patients was higher among inpatients than among patients in the emergency room and ICU patients. In the Rutschmann study, containing 123 emergency room cases, the proportion of PE in AE-COPD patients was estimated to be only 3.3% [15, 50]. In the Törk study, containing 36 emergency room cases, the proportion of PE in AE-COPD patients was estimated to be 36.1% [28]. There was a significant difference between these two studies, and the sample sizes of the included studies seemed to have a great impact on the results. As timely assessment of the possibility of PE was not made when emergency-room patients were admitted to the hospital, 33.5% of them had a delayed diagnosis [15, 50]. Furthermore, computed tomography pulmonary angiography (CTPA) for PE detection was not routinely performed in ICU patients, where the main concern is vital sign maintenance [51]. These variabilities explain the need for clinical studies, with a particular focus on the emergency room or ICU patients, to investigate the prevalence of PE in these patients with AE-COPD.

In our review, males, age, cigarette smoking, BMI, comorbidities (hypertension, HF, AF, IHD, diabetes, cancer) and immobilization did not increase the risk of PE in patients with AE-COPD, as indicated in Aleva *et al*.’s review [6]. While most research results indicate that surgery and cancer are related to PE, other clinical features, such as peripheral vascular disease, congestive heart failure, and worse exercise capacity, may also be associated with PE in COPD patients [14, 52]. Therefore, there are many potential reasons for the high incidence of PE in AE-COPD patients, which need to be further explored.

Early identification of PE among AE-COPD is of vital significance but difficult due to overlap in clinical symptoms. Patients with AE-COPD had an increased risk of PE when they had dyspnea, pleuritic chest pain, higher heart rate, lower limb asymmetry or edema and longer hospital times [24, 32, 33, 53], as supported by our study. Although four studies reported dyspnea in AE-COPD patients with or without PE [23, 25, 37, 53], three reported that all AE-COPD patients with or without PE had dyspnea [25, 37, 53]. Maritano *et al*. [23] suggested that the presence of isolated dyspnea had high sensitivity (92%) for the diagnosis of PE in those COPD patients who do not have increased cough and sputum. PE and AE-COPD are very similar, and the two are often indistinguishable clinically. Therefore, PE is easily neglected in patients with AE-COPD, resulting in delayed treatment and poor prognosis [14, 38]. Our study suggests that these clinical symptoms merit clinical attention and should be taken into account in patients with unexplained AE-COPD after excluding respiratory tract infection. Moreover, signs of cardiac dysfunction, such as hypotension, syncope, and right ventricular heart failure, can be used as diagnostic indicators of PE [32, 33, 53].

In our study, compared with patients with AE-COPD, patients with both PE and AE-COPD had higher D-dimer levels and WBC counts and lower PaO_2_ levels. Currently, there is still no specific biomarker for COPD complicated with PE. D-dimer testing is effective in the preliminary evaluation and management of patients with clinically suspected PE [54, 55]. However, because the blood of COPD patients is in a hypercoagulable state, the D-dimers of these patients are prone to false-positive results, and D-dimer cutoff values may need to be adjusted for such patients. Akpinar *et al*. [33] suggested that a a D-dimer cutoff value of 0.95 ng/L (sensitivity 70%, specificity 71%) was more accurate for the exclusion of PE in patients with AE-COPD than the conventional cutoff value (<0.5 ng/L). However, these cutoff values have not been prospectively validated. To date, the study of the relationships between blood biomarkers and the occurrence of PE in COPD patients is immature area. Wang *et al*. [38] discovered that the red blood cell distribution width standard deviation (RDW-SD) combined with D-dimer levels (sensitivity 87.5%, specificity 83.5%) may be useful in predicting the occurrence of PE in patients with COPD [56–58]. Bialas *et al*. [30] found that MLPR detection shows very high accuracy for predicting PE in patients with COPD (sensitivity 100%, specificity 85.7%), and Wang *et al*. [39] revealed that PDW is elevated in COPD patients with PE and associated with the risk of PE. Yang *et al*. [59] suggested that the activation of eosinophils increases the likelihood of thrombosis. However, it is unclear whether eosinophil activation markers can predict PE in patients with exacerbation of COPD.

Finally, in our analysis, the mortality of PE combined with AE-COPD was significantly higher than that of AE-COPD, especially in the ICU setting. Hassen *et al*. [25] found that PE is a common etiology of severe exacerbation of COPD and leads to high mortality and poor prognosis. The study had reported that multiple organ failure was more common in patients with both AE‑COPD and PE than those without PE [32]. The results of the international multi-center RIETE study showed that compared with patients with COPD, the 3-month mortality rate (10.8% vs. 7.6%), bleeding rate (4.5% vs. 2.3%) and recurrence rate of PE (1.5% vs. 1.1%) were all higher in patients with COPD combined with PE [60].

Although the prevalence of PE in AE-COPD patients has been reported in previous reviews [6, 8, 14, 15], our study included the relatively large sample size and more studies than these previous reviews. To better estimate of the prevalence of PE combined with AE-COPD, we performed subgroup analyses, and simultaneously included laboratory data, clinical features, and mortality analyses, which have not been included in previous studies but are important for recognizing PE with AE-COPD.

### Limitations

This study has several limitations. First, heterogeneity of the findings was observed among the included studies, with the prevalence rates of PE ranging from 3.3% to 36.1%; this range is wider than the previously reported range of 3.3%-26.6%. The inclusion of multiple studies resulted in a large amount of variability. To account for this heterogeneity, we used a random effects model in the meta-analysis. Moreover, the subgroup meta-analysis contributes to the understanding of PE prevalence in AE-COPD within this heterogeneous context. Second, the majority of the studies were conducted in Asia; there may be regional differences in PE prevalence. Finally, we admit that although we conducted a comprehensive literature search and employed strict data extraction, we may still miss some relevant studies.

## Conclusions

In summary, our meta-analysis revealed that the prevalence of PE in AE-COPD patients was 17.2%, with a range of 3.3% ~36.1%. When AE-COPD patients show dyspnea, pleuritic chest pain, higher heart rate, lower limb asymmetry or edema and longer hospital stays, we should be alert to the occurrence of PE. Additionally, compared with AE-COPD patients, AE-COPD patients with PE have higher D-dimer levels and WBC counts and lower PaO2 levels. These findings merit clinical attention. Our findings also highlight that PE is associated with higher mortality in severe COPD exacerbation patients during ICU admission. However, the accurate diagnosis and evaluation of AE-COPD with PE remains a great challenge for clinicians.

## Supporting information

S1 Checklist PRISMA 2009 checklist used in this meta-analysis. (DOC)

S1 Table The search strategy for articles on PE prevalence in AE-COPD patients. (DOC)

S2 Table The studies and characteristics included in the meta-analysis. (DOC)

S3 Table Subgroup analyses of the clinical characteristics of AE-COPD patients with PE or without PE. (DOCX)

S4 Table NOS scores. (DOC)

S1 Fig Forest plots of meta-analysis on the prevalence of DVT prevalence in AE-COPD patients. (TIF)

S2 Fig Subgroup analyses of laboratory parameters in AE-COPD patients with PE or without PE. (TIF)

## Abbreviations

95% CI: 95% confidence interval
AE-COPD: Acute exacerbation of chronic obstructive pulmonary disease
AF: Atrial fibrillation
BMI: Body mass index
CAD: Coronary heart disease
CT: Computed tomography scan
CTA: Computed tomography angiography
CTPA: Computed tomography pulmonary angiography
DVT: Deep vein thrombosis
ER: Emergency room
HF: Heart failure
ICU: Intensive care unit
IHD: Ischemic heart diseases
MD: Mean differences
MDCT: Multidetector helical computed tomography scan
NA: Not available
OR: Odds ratio
PE: Pulmonary embolism
