# Supplementary material for: The prevalence and clinical features of pulmonary embolism in patients with AE-COPD: A meta-analysis and systematic review
Source: PLoS One. 2021 Sep 2;16(9):e0256480. doi: 10.1371/journal.pone.0256480 (PMC8412363; doi:10.1371/journal.pone.0256480)
Supplement: S1 Table — (DOC) [file pone.0256480.s002.doc]

**S1 Table. The search strategy for articles on PE prevalence in AE-COPD patients**

**PubMed**

| **#** | **Searches** | **Results** |
| --- | --- | --- |
| **#1** | (pulmonary disease chronic obstructive) [Title/Abstract] OR (chronic obstructive pulmonary disease) [Title/Abstract] OR (chronic airflow obstruction) [Title/Abstract] OR COPD [Title/Abstract] OR (chronic obstructive airway disease) [Title/Abstract] OR COAD [Title/Abstract] OR (chronic obstructive lung disease) [Title/Abstract] OR (chronic airflow obstruction) [Title/Abstract] OR (obstructive respiratory disease) [Title/Abstract] |  |
| **#2** | (pulmonary embolism) [Title/Abstract] OR (pulmonary thromboembolism) [Title/Abstract] OR PE [Title/Abstract] OR (pulmonary emboli) [Title/Abstract] OR (pulmonary embolus) [Title/Abstract] OR (pulmonary microembolism) [Title/Abstract]) |  |
| **#3** | **#1 and #2** | **891** |

**Web of Science**

| **#** | **Searches** | **Results** |
| --- | --- | --- |
| **#1** | TS= ("pulmonary disease chronic obstructive" OR "chronic obstructive pulmonary disease" OR "chronic airflow obstruction" OR COPD OR "chronic obstructive airway disease" OR COAD OR "chronic obstructive lung disease" OR "chronic airflow obstruction" OR "obstructive respiratory disease") |  |
| **#2** | TS= ("pulmonary embolism" OR "pulmonary thromboembolism" OR PE OR "pulmonary emboli" OR "pulmonary embolus" OR "pulmonary microembolism") |  |
| **#3** | **#1 and #2** | **1372** |

**EMBASE**

| **#** | **Searches** | **Results** |
| --- | --- | --- |
| **#1** | ('pulmonary disease chronic obstructive'/exp OR 'chronic obstructive pulmonary disease'/ab,ti OR COPD OR 'chronic airflow obstruction'/ab,ti OR COAD OR 'chronic obstructive airway disease'/ab,ti OR 'chronic obstructive lung disease'/ab,ti OR 'chronic airflow obstruction'/ab,ti OR 'obstructive respiratory disease'/ab.ti) |  |
| **#2** | ('pulmonary embolism'/exp OR ' pulmonary thromboembolism '/ab,ti OR PE OR 'pulmonary emboli'/ab,ti OR ‘pulmonary embolus’/ab,ti OR 'pulmonary microembolism'/ab,ti) |  |
| **#3** | **#1 and #2** | **3208** |

**Cochrane Library**

| **#** | **Searches** | **Results** |
| --- | --- | --- |
| **#1** | ((pulmonary disease chronic obstructive) OR (chronic obstructive pulmonary disease) OR (chronic airflow obstruction) OR COPD OR (chronic obstructive airway disease) OR (chronic obstructive lung disease) OR (chronic airflow obstruction) OR (obstructive respiratory disease)) |  |
| **#2** | ((pulmonary embolism) OR (pulmonary thromboembolism) OR PE OR (pulmonary emboli) OR (pulmonary embolus) OR (pulmonary microembolism)) |  |
| **#3** | **#1 and #2** | **144** |
