# Supplementary material for: The prevalence and clinical features of pulmonary embolism in patients with AE-COPD: A meta-analysis and systematic review
Source: PLoS One. 2021 Sep 2;16(9):e0256480. doi: 10.1371/journal.pone.0256480 (PMC8412363; doi:10.1371/journal.pone.0256480)
Supplement: S2 Table — (DOC) [file pone.0256480.s003.doc]

**S2 Table. The studies and characteristics included in the meta-analysis**

| **Author, Year** | **Country** | **Setting** | **Patient selection criteria** | **Patient exclusion criteria** | **Sample size** | **Age** ^a^ | **NO. of**  **Male** | **NO. of**  **PE** | **NO. of**  **DVT** | **PE confirmed method** | **NOS** |
| --- | --- | --- | --- | --- | --- | --- | --- | --- | --- | --- | --- |
| Maritan Furcada J,2020^1^ | Argentina | NA | AE-COPD patients were suspected PE | NA | 168 | 74 | 106 | 37 | NA | CTA | 7 |
| Jindal A,2020^2^ | Indian | Inpatients | Patients with AE-COPD of unknown origin |  | 110 | NA | 77 | 20 | 6 | CTPA | 6 |
| Dentali F,2020^3^ | Italian | Inpatients | AE-COPD patients were suspected PE | NA | 1043 | 75.9 | 683 | 132 | 88 | CTA | 7 |
| Hassen MF,2019^4^ | Tunisia | ICU | AE-COPD patients require mechanical ventilation |  | 131 | 68 | 104 | 18 | 1 | MDCT | 8 |
| Jing X,2018^5^ | China (Henan) | NA | Patients with hemodynamically stable AECOPD |  | 419 | NA | 274 | 96 | NA | CTPA | 7 |
| Davoodi M,2018^6^ | Iran | NA | Patients with AE-COPD |  | 68 | NA | 33 | 5 | NA | CTPA | 7 |
| Bialas AJ,2018^7^ | Poland | Inpatients | AE-COPD patients were suspected PE |  | 101 | 72 | 56 | 24 | 7 | CTPA | 8 |
| Törk M,2017^8^ | Turkey | ER | Patients with AE-COPD | NA | 36 | 71.9 | 29 | 13 | NA | CTPA | 6 |
| AbdelHalim HA,2017^9^ | Egypt | Inpatients | Patients with AE-COPD |  | 83 | 56.2 | 83 | 11 | NA | CTPA | 7 |
| Shapira-Rootman M,2015^10^ | Israel | Inpatients | Patients with AE-COPD |  | 49 | 65.5 | 35 | 9 | 0 | CTPA | 7 |
| Bahloul M,2015^11^ | Tunisia | ICU | Patients were admitted to the ICU because of AE-COPD | NA | 131 | 68.6 | 118 | 23 | NA | CT | 6 |
| Akpinar EE,2014^12^ | Turkey | Inpatients | Patients with AE-COPD |  | 172 | 71.3 | 142 | 50 | 50 | CTA | 8 |
| Choi K-J,2013^13^ | South Korea | Inpatients | Patients with AE-COPD |  | 103 | 71 | 70 | 5 | 4 | CTPA | 6 |
| Kamel MM,2013^14^ | Egypt | Inpatients | Patients with AE-COPD | NA | 105 | 49.3 | 105 | 30 | 11 | CTPA | 8 |
| Gunen H,2010^15^ | Turkey | Inpatients | Patients with AE-COPD of unknown origin |  | 131 | 67.1 | 97 | 18 | 14 | CTA | 8 |
| Rutschmann OT,2007^16^ | Switzerland | ER | Patients with AE-COPD |  | 123 | 71 | 84 | 4 | 2 | MDCT | 7 |
| Tillie-Leblond I,2006^17^ | France | Inpatients | Patients with AE-COPD of unknown origin and did not require invasive mechanical  ventilation |  | 197 | 60.5 | 165 | 49 | 25 | CTA | 7 |

^a^ mean or median;

Patient exclusion criteria: Patients with other causes of respiratory deterioration (eg, pneumothorax, pneumonia, pleural effusion, pulmonary edema, or iatrogenic factor);Patients are allergy to intravenous contrast medium; Patients with long term anticoagulant therapy; Patients with undergoing mechanical or noninvasive ventilation; Patients are unable to complete informed consent because of various reasons or not give consent; Patients with renal diseases; Patients with other concurrent diseases such as any heart disease, hypertension, diabetes, or hepatic disease etc.; Patients with active cancer, surgery within the last months; Patients with any myeloproliferative disorder, myelofibrosis, Glanzmann thrombasthenia, May–Hegglin anomaly, Bernard–Soulier syndrome, suspicion of disseminated intravascular coagulation, blood transfusion in the last 2 months; Patients were not inability to perform spirometry; Patients were a hypercoagulable state

**References**

1. Maritano Furcada J, Castro HM, De Vito EL, Grande Ratti MF, Posadas-Martinez ML, Giunta D, et al. Diagnosis of Pulmonary Embolism in Patients with Acute Exacerbations of Chronic Obstructive Pulmonary Disease: a Cross-sectional Study. The clinical respiratory journal, 2020.14(12):1176-1181.

2. Jindal A, Rathore YS, Joshi V, Jain S, Khippal N. A cross-sectional study for the evaluation of pulmonary embolism in unexplained dyspnea in acute exacerbation of chronic obstructive pulmonary disease. Indian Journal of Respiratory Care 2020; 9(2): 191-5.

3. Dentali F, Pomero F, Micco PD, La Regina M, Landini F, Mumoli N, et al. Prevalence and risk factors for pulmonary embolism in patients with suspected acute exacerbation of COPD: a multi-center study. Eur J Intern Med, 2020. 80: p. 54-59.

4. Hassen MF, Tilouche N, Jaoued O, Elatrous S. Incidence and Impact of Pulmonary Embolism During Severe COPD Exacerbation. Respiratory care 2019; 64(12): 1531-6.

5. Jing X, Zhang G, Zhang B, Dai L, Wang X, Jia L, et al. Efficacy and safety of low-dose urokinase for the treatment of hemodynamically stable AECOPD patients with acute pulmonary thromboembolism. Clinical Respiratory Journal, 2018. 12(5): p. 1882-1890.

6. Davoodi M, Rezvankhah B, Moghadam KG, Taheri APH. The prevalence and predicting factors of pulmonary thromboembolism in patients with exacerbated chronic obstructive pulmonary disease. Advances in Respiratory Medicine, 2018. 86(4): p. 168-171.

7. Bialas AJ, Kornicki K, Ciebiada M, Antczak A, Sitarek P, Milkowska-Dymanowska J, et al. Monocyte to large platelet ratio as a diagnostic tool of pulmonary embolism in patients with acute exacerbation of COPD. Polish Archives of Internal Medicine, 2018. 128(1): p. 906-914.

8. Törk M, Yildirim F, Sevimli N, Köktörk N. Impact of pulmonary embolism on acute chronic obstructive pulmonary disease exacerbation. Gazi Medical Journal, 2017. 28(3): p. 168-170.

9. AbdelHalim HA and HH AboElNaga. Acute exacerbation of COPD with pulmonary embolism: A new D-dimer cut-off value. Egyptian Journal of Chest Diseases and Tuberculosis, 2017. 66(2): p. 227-230.

10. Shapira-Rootman M, Beckerman M, Soimu U, Nachtigal A, Zeina AR. The prevalence of pulmonary embolism among patients suffering from acute exacerbations of chronic obstructive pulmonary disease. Emerg Radiol, 2015. 22(3): p. 257-60.

11. Bahloul M, Chaari A, Tounsi A, Baccouche N, Abid H, Chtara K, et al. Incidence and impact outcome of pulmonary embolism in critically ill patients with severe exacerbation of chronic obstructive pulmonary diseases. Clinical Respiratory Journal, 2015. 9(3): p. 270-277.

12. Akpinar EE, Hoşgün D, Akpinar S, Ataç GK, Doğanay B, Gülhan M. Incidence of pulmonary embolism during COPD exacerbation. Jornal brasileiro de pneumologia : publicacao oficial da Sociedade Brasileira de Pneumologia e Tisilogia 2014; 40(1): 38-45.

13. Choi K-J, Cha S-L, Shin K-M, Lee J, Hwangbo Y, Yoo S-S, et al. Prevalence and Predictors of Pulmonary Embolism in Korean Patients with Exacerbation of Chronic Obstructive Pulmonary Disease. Respiration, 2013. 85(3): p. 203-209.

14. Kamel MM, Moussa H, Ismail A. Prevalence of venous thrombo-embolism in acute exacerbations of chronic obstructive pulmonary disease. Egyptian Journal of Chest Diseases and Tuberculosis 2013; 62(4): 557–66.

15. Gunen H, Gulbas G, In E, Yetkin O, Hacievliyagil SS. Venous thromboemboli and exacerbations of COPD. European Respiratory Journal 2010; 35(6): 1243-8.

16. Rutschmann OT, Cornuz J, Poletti P-A, Bridevaux P-O, Hugli OW, Qanadli SD, et al., Should pulmonary embolism be suspected in exacerbation of chronic obstructive pulmonary disease? Thorax, 2007. 62(2): p. 121-125.

17. Tillie-LI, Marquette CH, Perez T, Scherpereel A, Zanetti C, Tonnel AB, et al. Pulmonary embolism in patients with unexplained exacerbation of chronic obstructive pulmonary disease: Prevalence and risk factors. Annals of Internal Medicine, 2006. 144(6): p. 390-396.
