# Supplementary material for: The prevalence and clinical features of pulmonary embolism in patients with AE-COPD: A meta-analysis and systematic review
Source: PLoS One. 2021 Sep 2;16(9):e0256480. doi: 10.1371/journal.pone.0256480 (PMC8412363; doi:10.1371/journal.pone.0256480)
Supplement: S3 Table — (DOCX) [file pone.0256480.s004.docx]

**S3 Table. Subgroup analyses of the clinical characteristics of AE-COPD patients with PE or without PE**

| Variables | No. of  studies | No. of  patients | OR/MD | 95% CI | *P* | Heterogeneity | | | Model used |
| --- | --- | --- | --- | --- | --- | --- | --- | --- | --- |
|  |  |  |  |  |  | I^2^ (%) | *P_H_* | |  |
| Gender-female | **11** | **777** | **1.33** | **1.06-1.67** | **0.01** | **39.4%** | | **0.09** | **Fixed model** |
| Age | 11 | 2482 | 1.70 | -0.13-3.53 | 0.07 | 62.3% | | <0.01 | Random model |
| Smoking  (pack-year) | 4 | 438 | -1.70 | -23.56-20.15 | 0.88 | 86.6% | | <0.01 | Random model |
| BMI | 3 | 307 | 0.31 | -1.11-1.74 | 0.67 | 0.0% | | 0.69 | Fixed model |
| **Co-morbidities** |  |  |  |  |  |  | |  |  |
| Hypertension | 7 | 533 | 1.28 | 0.97-1.69 | 0.07 | 15.1% | | 0.31 | Fixed model |
| HF | 6 | 259 | 1.33 | 0.94-1.89 | 0.11 | 28.0% | | 0.23 | Fixed model |
| AF | 3 | 132 | 1.43 | 0.89-2.30 | 0.14 | 23.7% | | 0.27 | Fixed model |
| IHD | 3 | 42 | 2.19 | 0.27-17.26 | 0.77 | 71.4% | | 0.03 | Random model |
| Diabetes | 6 | 280 | 0.88 | 0.61-1.28 | 0.51 | 0.0% | | 0.50 | Fixed model |
| Cancer | 7 | 362 | 1.62 | 0.82-3.19 | 0.17 | 69.1% | | <0.01 | Random model |
| Immobilization | 3 | 275 | 2.74 | 0.90-8.29 | 0.07 | 60.8% | | 0.07 | Random model |
| **Characteristics** |  |  |  |  |  |  | |  |  |
| Heart rate | **4** | **343** | **20.51** | **4.94-36.08** | **<0.01** | **94%** | | **<0.01** | **Random model** |
| Dyspnea | **1** | **168** | **6.77** | **1.97-23.22** | **<0.01** | **-** | | **-** | **Fixed/ Random model** |
| Cough | **3** | **321** | **0.65** | **0.12-3.66** | **0.83** | **88.2%** | | **<0.01** | **Random model** |
| Pleuritic chest pain | **4** | **191** | **3.25** | **2.06-5.12** | **<0.01** | **0.0%** | | **0.52** | **Fixed model** |
| Hemoptysis | 3 | 34 | 4.92 | 0.61-39.63 | 0.13 | 81.4% | | <0.01 | Random model |
| Lower limb asymmetry or edema | **3** | **101** | **2.46** | **1.51-4.00** | **<0.01** | **25.1%** | | **0.26** | **Fixed model** |
| Hospital stays (days) | **6** | **701** | **3.66** | **3.01-4.31** | **<0.01** | **42%** | | **0.13** | **Fixed model** |
