# Supplementary material for: The prevalence and clinical features of pulmonary embolism in patients with AE-COPD: A meta-analysis and systematic review
Source: PLoS One. 2021 Sep 2;16(9):e0256480. doi: 10.1371/journal.pone.0256480 (PMC8412363; doi:10.1371/journal.pone.0256480)
Supplement: S4 Table — (DOC) [file pone.0256480.s005.doc]

**S4 Table. NOS scores**

| **Author** | **1) Define the source of information (survey, record review)** | **2) List inclusion and exclusion criteria for exposed and unexposed subjects (cases and controls) or refer to previous publications** | **3) Indicate time period used for identifying patients** | **4) Indicate whether or not subjects were consecutive if not population-based** | **5) Indicate if evaluators of subjective components of study were masked to other aspects of the status of the participants** | **6) Describe any assessments undertaken for quality assurance purposes (e.g., test/retest of primary outcome measurements)** | **7) Explain any patient exclusions from analysis** | **8) Describe how confounding was assessed and/or controlled** | **9) If applicable, explain how missing data were handled in the analysis** | **10) Summarize patient response rates and completeness of data collection** | **11) Clarify what follow-up, if any, was expected and the percentage of patients for which incomplete data or follow-up was obtained** | **Score** |
| --- | --- | --- | --- | --- | --- | --- | --- | --- | --- | --- | --- | --- |
| Maritan Furcada J,2020^1^ | 1 | 1 | 1 | 1 | 0 | 0 | 1 | 0 | 1 | 1 | 0 | 7 |
| Jindal A,2020^2^ | 1 | 1 | 0 | 1 | 0 | 0 | 1 | 0 | 1 | 1 | 0 | 6 |
| Dentali F,2020^3^ | 1 | 1 | 1 | 1 | 0 | 0 | 1 | 0 | 1 | 1 | 0 | 7 |
| Hassen MF,2019^4^ | 1 | 1 | 1 | 1 | 0 | 0 | 1 | 0 | 1 | 1 | 0 | 7 |
| Jing X,2018^5^ | 1 | 1 | 1 | 1 | 0 | 0 | 1 | 0 | 1 | 1 | 1 | 8 |
| Davoodi M,2018^6^ | 1 | 1 | 1 | 1 | 0 | 0 | 1 | 0 | 1 | 1 | 0 | 7 |
| Bialas AJ,2018^7^ | 1 | 1 | 1 | 1 | 0 | 0 | 1 | 1 | 1 | 1 | 0 | 8 |
| Törk M,2017^8^ | 1 | 1 | 1 | 1 | 0 | 0 | 0 | 0 | 1 | 1 | 0 | 6 |
| AbdelHalim HA,2017^9^ | 1 | 1 | 1 | 1 | 0 | 0 | 1 | 0 | 1 | 1 | 0 | 7 |
| Shapira-Rootman M,2015^10^ | 1 | 1 | 1 | 1 | 0 | 0 | 1 | 0 | 1 | 1 | 0 | 7 |
| Bahloul M,2015^11^ | 1 | 1 | 1 | 1 | 0 | 0 | 0 | 0 | 1 | 1 | 0 | 6 |
| Akpinar EE,2014^12^ | 1 | 1 | 1 | 1 | 0 | 1 | 1 | 1 | 1 | 0 | 0 | 8 |
| Choi K-J,2013^13^ | 1 | 1 | 1 | 1 | 0 | 0 | 1 | 0 | 0 | 1 | 0 | 6 |
| Kamel MM,2013^14^ | 1 | 1 | 1 | 1 | 0 | 0 | 1 | 1 | 1 | 1 | 0 | 8 |
| Gunen H,2010^15^ | 1 | 1 | 1 | 1 | 0 | 0 | 1 | 1 | 1 | 1 | 0 | 8 |
| Rutschmann OT,2007^16^ | 1 | 1 | 1 | 0 | 0 | 0 | 1 | 0 | 1 | 1 | 1 | 7 |
| Tillie-Leblond I,2006^17^ | 1 | 1 | 1 | 1 | 0 | 0 | 1 | 0 | 1 | 1 | 0 | 7 |
