# Supplementary figures and images for: The prevalence and clinical features of pulmonary embolism in patients with AE-COPD: A meta-analysis and systematic review
Source: PLoS One. 2021 Sep 2;16(9):e0256480. doi: 10.1371/journal.pone.0256480 (PMC8412363; doi:10.1371/journal.pone.0256480)

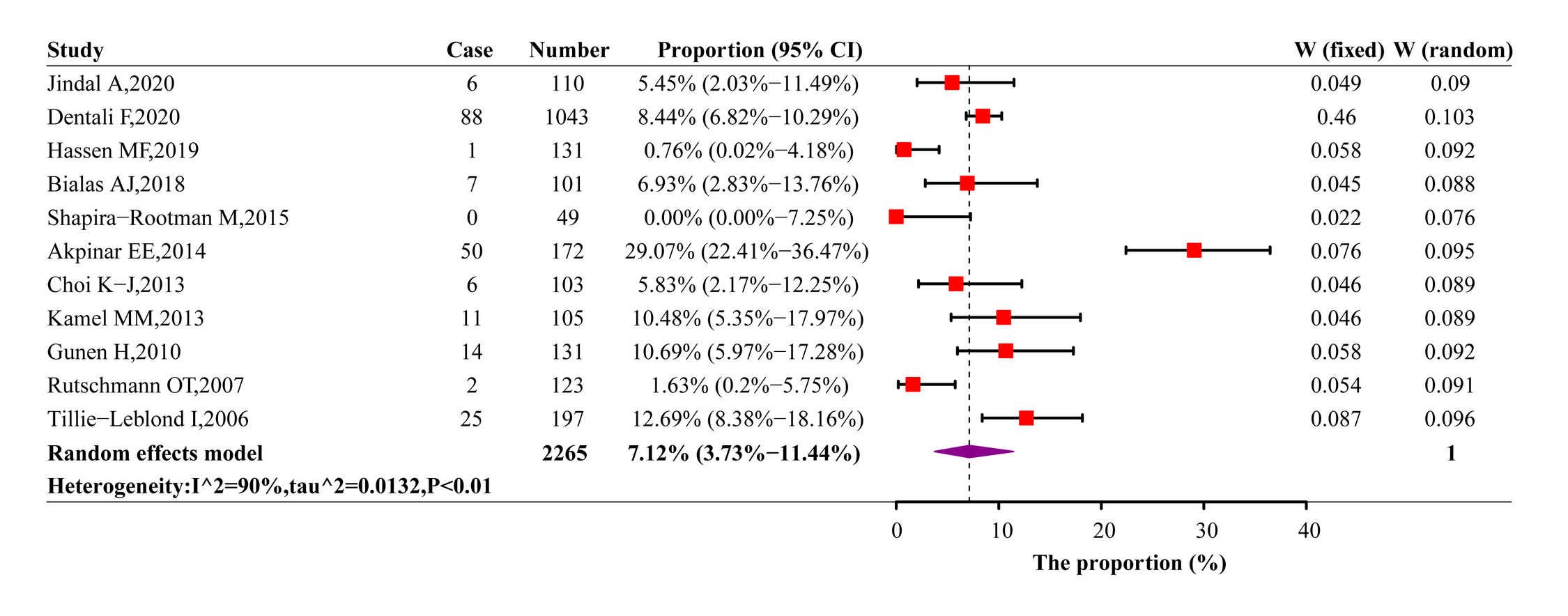

Supplement: S1 Fig — (TIF) [file pone.0256480.s006.tif]

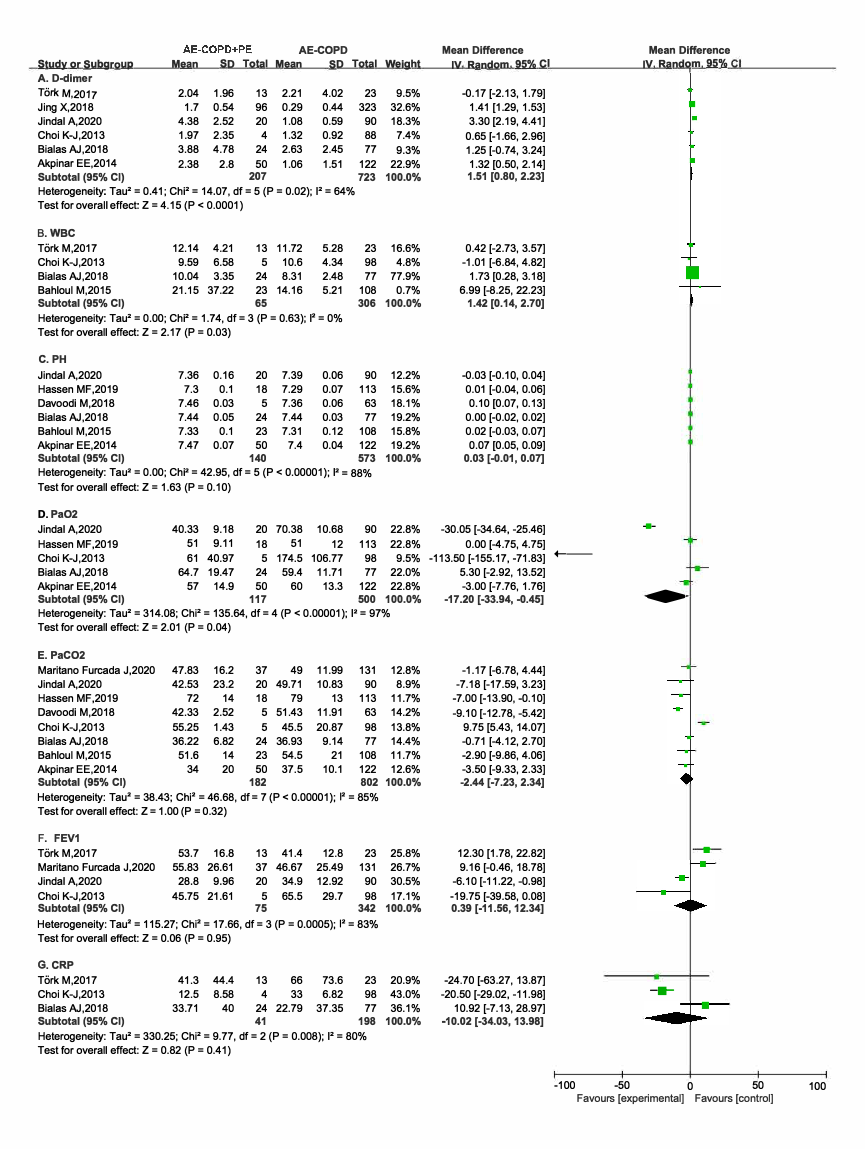

Supplement: S2 Fig — (TIF) [file pone.0256480.s007.tif]
